# Metabolomics and Transcriptomics Jointly Explore the Mechanism of Pod Color Variation in Purple Pod Pea

**DOI:** 10.3390/cimb47020094

**Published:** 2025-02-01

**Authors:** Xiaojuan Zhong, Mei Yang, Xiaoyan Zhang, Yuanfang Fan, Xianshu Wang, Chao Xiang

**Affiliations:** 1 Crop Research Institute of Sichuan Academy of Agricultural Sciences/Environment-Friendly Crop Germplasm Innovation and Genetic Improvement Key Laboratory of Sichuan Province, Chengdu 610066, China; zhongxj@scsaas.cn (X.Z.); yangmeitong2012@163.com (M.Y.); yuanfang518@scsaas.cn (Y.F.); wangxs1323690423@163.com (X.W.); 2 Qingdao Academy of Agricultural Sciences, Qingdao 266100, China; zxysea@126.com

**Keywords:** *Pisum sativum* L., pod color, metabolome, transcriptome, flavonoid metabolism, anthocyanin

## Abstract

Although the pod color was one of the seven characteristics Mendel studied in peas, the mechanism of color variation in peas with purple pods has not been reported. This study systemically analyzed the difference between two pea accessions with green pods (GPs) and purple pods (PPs) at two pod developmental stages from the metabolome and transcriptome levels, aiming to preliminarily explore the mechanism and of color variation in PPs and screen out the candidate genes. A total of 180 differentially accumulated metabolites (DAMs) belonged to seven flavonoid subgroups and 23 flavonoid-related differentially expressed genes (DEGs) were identified from the analysis of the KEGG (Kyoto Encyclopedia of Genes and Genomes) pathway enrichment, respectively. Among the 180 flavonoid metabolites, ten anthocyanin compounds, which were the principal pigments in PPs and might be the major reason for the purple color formation, were significantly up-accumulated in both of the different pod development stages of PPs. A transcriptome analysis revealed that eight genes encoding enzymes (C4H, CHI, F3H, F3’H, F3’5’H, DFR, ANS, and FLS) involved in the flavonoid synthesis pathway were significantly upregulated in PPs and finally resulted in the significant accumulation of flavonoid and anthocyanin metabolites. The joint analysis of two omics and a weighted gene co-expression network analysis (WGCNA) also screened out that the WD-40 protein-encoding gene, one WRKY and three MYB transcription factor genes exhibited significant upregulation in PPs, and highly correlated with several structural genes in flavonoid synthesis pathways, indicating that these genes are involved in the regulation of pod color formation in PPs. Overall, the results of this study first explored the mechanism underlying the purple color variation between PPs and GPs, and then preliminarily screened out some candidate genes responsible for the pod color formation in PPs.

## 1. Introduction

The pea (*Pisum sativum* L.) is an important legume crop and a primary source of plant-based protein, starch, and fiber for both humans and animals [1]. Because of its biological nitrogen fixation capacity, the pea is both a vegetable and an arable crop. As a domesticated legume crop, peas have been cultivated for more than 10,000 years [2,3]. According to the Food and Agriculture Organization (FAO; https://www.fao.org/faostat/ (accessed on 20 December 2024)), the harvested area of peas ranks fourth among legumes. The pea is also the research foundation of Mendelian genetics [4]. 

The pod color was one of the seven characteristics Mendel investigated in peas. Three different pod colors are found in peas, viz., green, yellow, and purple. In previous research on yellow pods [4], the *GP* locus (pod color) on chromosome 3 was considered the key locus responsible for pod color trait. A more recent study analyzed the DNA sequences of the *GP* locus at the genomic location and transcriptome profiles of green and yellow pod lines, and genes (*Psat0s4355g0080* and *Psat0s4355g0120*) encoding 3’ exoribonucleases were identified as potential candidates controlling pod color [5]. The homologous gene of *Psat0s4355g0080*, *Pisum03G256280*, with one SNP variation in its promoter was detected exclusively in accessions showing yellow pod coloration [6]. A recent study showed that a defection in the chlorophyll synthase gene (*ChlG*) generated aberrant transcripts and mediated the yellow pod phenotype of *gp* mutants [7]. All these studies show that there is a certain research for the key genes responsible for color variation in yellow pods. Nevertheless, there have been no related studies on color variation in peas with purple pods. 

The purple tissue of plants is generally rich in anthocyanins, which are one of the most important flavonoid compounds and a group of naturally occurring pigments responsible for the red–blue color [8,9]. The synthesis of anthocyanins was based on phenylalanine as a precursor, which was catalyzed by a series of enzymes to form stable anthocyanin in the cytoplasm and transported to the vacuole for enrichment. The structural genes responsible for anthocyanin biosynthesis included PAL (phenylalanine ammonialyase), C4H (cinnamate 4-hydroxylase), 4CL (4-coumarate: CoA ligase), CHS (chalcone synthase), CHI (chalcone isomerase), F3H (flavanone 3-hydroxylase), F3ʹH (flavonoid 3ʹ-hydroxylase), F3ʹ5ʹH (flavonoid 3ʹ5ʹ-hydroxylase), DFR (dihydroflavonol-4-reductase), and ANS (anthocyanin synthase). Anthocyanin biosynthesis was not only influenced by the structural genes but also precisely regulated by transcription factors at the transcription level. Numerous transcription factors related to anthocyanin synthesis have been currently identified, including MYB, bHLH, WD40, bZIP, NAC, WRKY, and MADS, among which R2R3-MYB, bHLH, and WD40 are the major regulators of anthocyanin synthesis. In most cases, these three transcription factors act as the R2R3-MYB-bHLH-WD40/WDR (MBW) complex, which has been investigated in several species. The pea line with the purple pod is a special germplasm resource material. No study has investigated the difference between the composition and genetic mechanism in flavonoids and the anthocyanin metabolic pathways between purple and green pods yet.

Pod color is an important agronomic trait of peas. Purple pod peas are not only rare in color, but also rich in antioxidants, which makes them more nutritious than ordinary green pod peas. The analysis and excavation of coloring material basis and related genes of purple pod peas can effectively explore and utilize these specific germplasm resources. However, the formation mechanism and molecular mechanism of purple pod pea has not been reported so far. This study systematically studied the metabolic and transcriptional levels of purple pod and green pod peas at two growth and development stages. Metabolome sequencing data were used to analyze the accumulation and metabolic mechanism pathways of pod color substances in purple pod peas. Combined with transcriptome data, the key genes that control the pod color of purple pod peas were analyzed and screened. This study provided candidate genes and specific gene resources for in-depth research on pod color formation and the directional improvement of pod color in peas, respectively.

## 2. Materials and Methods

### 2.1. Plant Materials

In this study, two pea accessions with green pods (GPs) and purple pods (PPs) selected from the F3 hybrid progeny of “Wuxu 1” (green pod) and W023 (purple pod) were used to reduce genetic background interference [10]. All plants were grown in an experimental field. The pods of GPs and PPs were collected on the fifth and tenth days of development after flowering (the first day was tagged as the day that the blossoms entirely opened [11]), respectively. In this study, six biological replicates were used at each stage for metabolomics analysis, and three biological replicates were used for RNA-Seq analysis and real-time quantitative PCR (qRT-PCR). Each sample were frozen in liquid nitrogen and stored at −80℃ for subsequent analysis. 

### 2.2. Metabolite Detection and Metabolomic Data Analysis

Metabolite extraction, liquid chromatography–mass spectrometry (LC-MS), and metabolomic data analysis were performed as reported previously [12]. Briefly, each sample was ground, sonicated, and extracted overnight in the extract solution and then filtered through a microfilter for LC-MS analysis. The LC-MS analysis was performed using an ACQUITY UPLCI-Class machine connected to a VIONIMSQTOF mass spectrometer (Waters Corporation, Milford, CT, USA) in both electrospray ionization (ESI) positive and negative ion modes. The data acquisition was performed in the full scan mode (*m*/*z* range 100–1200) and ESI mode.

Raw LC-MS data were subjected to baseline filtering, peak identification, integration, retention time correction, peak alignment, and normalization using the Progenesis QIV2.3 (Nonlinear Dynamics, Newcastle, UK) program before further analysis. The chemical identification of the compounds was completed by comparison with the Human Metabolome Database, Lipidmaps (V2.3), Metlin, and self-built databases, and the content of the compounds was calculated by combining the data of positive and negative ions to quantify the composite data matrix. Principal component analysis (PCA), orthogonal partial least squares discriminant analysis (OPLS-DA), and partial least squares discriminant analysis (PLS-DA) were performed on the data matrix using R. The variable importance of projection (VIP) yielded by the OPLS-DA model was used to evaluate the total contribution of each variable to the group. Based on the two-tailed Student’s *t*-test, differential metabolites with log2|FC| > 1, *p* < 0.05, and VIP > 1 were selected as significantly differentially accumulated metabolites (DAMs).

### 2.3. RNA Isolation, Sequencing and Bioinformatics Analysis

The total RNA of each sample was extracted using the TRIzol reagent (Invitrogen, Carlsbad, CA, USA). The purity, quantity, and integrity of RNA were evaluated using the Agilent 2100 Bioanalyzer (Agilent Technologies, Santa Clara, CA, USA) and NanoDrop 2000 spectrophotometer (Thermo Fisher Scientific, Waltham, MA, USA). The construction of a sequencing library and sequencing were performed using the VAHTS Universal V6 RNA-seq Library Prep Kit (Vazyme, Nanjing, China) and Ilumina Novaseq 6000 platform (Illumina, San Diego, CA, USA) respectively. More than 47 Gb paired-end reads were generated from 12 RNA-seq libraries. Filtered high-quality reads were mapped to the reference genome (EnsemblPlants-Pisum_sativum_v1a) using the TopHat2 in the HISAT2 software (http://ccb.jhu.edu/software/hisat2/index.shtml accessed on 20 December 2024).

The fragments per kilobase per million (FPKM) were used to represent the expression level of each gene. The DESeq 2 software (version 1.4.5) was used to screen the differentially expressed genes (DEGs) under the threshold of *p* < 0.05 and log2|FoldChange| > 1. The functional analysis of DEGs, including GO terms and KEGG pathway enrichment, were performed using the Blast2GO software (version 3.0.8) and KEGG Automatic Annotation Server, respectively.

### 2.4. Real-Time Quantitative Polymerase Chain Reaction (RT-qPCR) Validation

The expression levels of 12 genes involved in flavonoid biosynthesis pathway were selected randomly for validating the RNA-seq data by RT-qPCR. Each sample of cDNA was synthesized using the Maxima Reverse Transcriptase kit (EP0743, Thermo Fisher Scientific, Waltham, MA, USA), and RT-qPCR was carried out with the ChamQ Universa SYBR qPCR Master Mix kit (Vazyme, Nanjing, China). The amplification was performed using the 2X SG Fast qPCR Master Mix (B639271, BBI, Roche, Rotkreuz, Switzerland) and LightCycler480 II (Roche, Rotkreuz, Switzerland) with the *TubA1* (U12589.1) and *GAPDH* (U34988.1) genes in pea as internal reference genes. The relative expression levels of the genes were calculated using the 2^−∆∆Ct^ method.

### 2.5. Weighted Gene Co-Expression Network Analysis (WGCNA)

The screened DEGs and DAMs were applied to build a regulatory network through weighted gene co-expression network analysis (WGCNA) tools on the OECloud platform (https://cloud.oebiotech.com accessed on 20 December 2024). The power value (weight parameter of adjacency matrix) was selected as 30 to establish a weighted co-expression network model. The correlation coefficients were also calculated between the hub genes in the modules and the differential metabolites, and the top 50 genes with the highest connectivity in each module were used to construct the core gene network.

## 3. Results

### 3.1. Metabolome Analysis of Green and Purple Pods

**Figure 1:**
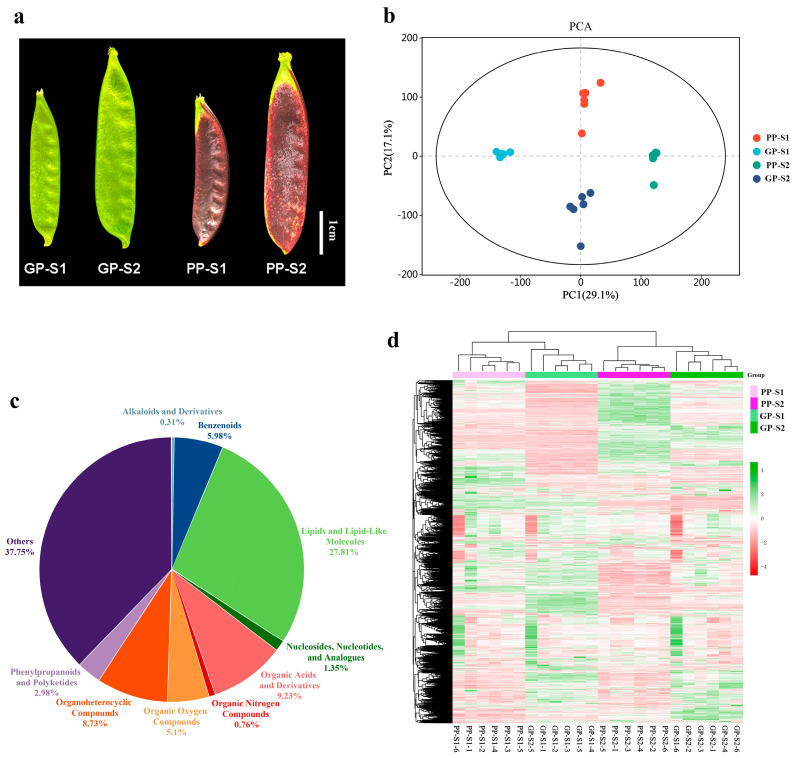
Metabolism data of the two pea accessions of green pods (GPs) and purple pods (PPs). (**a**). Morphology of GPs and PPs at two stages. S1: the pods at five days after anthesis; S2: the pods at ten days after anthesis; bar =1 cm. (**b**). Principal component analysis (PCA) of all samples. (**c**). Classification statistics of all metabolites identified in all samples. (**d**). Heatmap showing the variation in all differentially accumulated metabolites (DAMs) in all samples. The data used for the heatmap were horizontally normalized; red represents relatively high content, and green represents relatively low content.

Compared with the pea line with the green pod (GP), the purple pod (PP) accession exhibited intense purple pigments on the pod skin during the developmental stages (Figure 1a). The pods of GPs and PPs at five (S1) and ten (S2) days after anthesis, representing the early and rapid development stage of pod growth, respectively, were collected for multiomics testing and analysis. A PCA was conducted on all of the samples used for metabolome analysis to detect the overall metabolic differences between the groups and the degree of variation between the samples within a group (Figure 1b). The results of the PCA showed that although the first (PC1) and second (PC2) principal components could explain only 29.1% and 17.1% of the total variance, respectively, all of the samples could be clearly divided into four groups based on different pea accessions and different pod development stages, and the within-group variation in each sample was small, indicating that each sample was repeatable. Based on the LC-MS detection platform, 14,975 metabolites were identified (Table S1), including 27.81% of lipids and lipid-like molecules, 9.23% of organic acids and derivatives, 8.73% of organoheterocyclic compounds, 5.98% of benzenoids, 5.1% of organic oxygen compounds, 2.98% of phenylpropanoids and polyketides, 1.35% of nucleosides, nucleotides, and analogs, 0.76% of organic nitrogen compounds, 0.31% of alkaloids and derivatives, and 37.75% of others (Figure 1c). The DAMs between pairwise comparisons were screened based on the threshold of log2|FC| > 1, *p* < 0.05, and VIP > 1, and the hierarchical cluster analysis of all of the DAMs revealed that four clear clusters were classified between the green and purple pea lines and the two developmental stages of pods, indicating that the metabolites between the green and purple pea lines exhibited different accumulation levels (Figure 1d).

**Figure 2:**
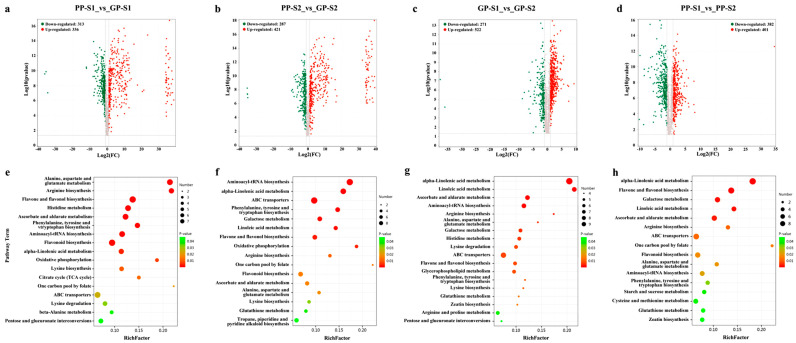
Statistics and function analysis of DAMs between GPs and PPs at two developmental stages. (**a**–**d**). Volcano maps of upregulated and downregulated differential metabolites in each comparison group. (**e**–**h**). The KEGG pathway enrichment of all DAMs in each comparison group. The ordinate represents the pathway term, and the abscissa represents the rich factor. The sizes of the dots represent the different number of DAMs, and the colors of the dots represent different *p* values.

In this study, the pairwise comparisons included PP-S1_vs_GP-S1, PP-S2_vs_GP-S2, GP-S1_vs_GP-S2, and PP-S1_vs_PP-S2, wherein the former was the comparison group, and the latter was the control group in each pairwise comparison. Among these comparisons, there were 649 DAMs (313 downregulated and 336 upregulated) in the PP-S1_vs_GP-S1 comparison, 708 DAMs (287 downregulated and 421 upregulated) in the PP-S2_vs_GP-S2 comparison, 793 DAMs (271 downregulated and 522 upregulated) in the GP-S1_vs_GP-S2 comparison, and 783 DAMs (382 downregulated and 401 upregulated) in the PP-S1_vs_PP-S2 comparison (Figure 2a–d). Table S2 shows the detailed information of all DAMs in each comparison. For the two varieties, the purple pod had more upregulated DAMs than the green pods at the two developmental stages (Figure 2a,b). However, in the comparisons between the S1 and S2 stages for the same variety, the purple pods had more downregulated metabolites and fewer upregulated metabolites compared with the GP materials (Figure 2c,d). 

Next, the KEGG pathway enrichment analysis of DAMs in each comparison was performed to identify the major biochemical pathways (Table S3 and Figure 2e–h). Several pathways were enriched in all groups, including the alanine, aspartate, and glutamate metabolism; ABC transporters; alpha-linolenic acid metabolism; aminoacyl-tRNA biosynthesis; arginine biosynthesis; ascorbate and aldarate metabolism; flavone and flavonol biosynthesis; and phenylalanine, tyrosine, and tryptophan biosynthesis (Figure 2e–h). The purple pods had more DAMs enriched in the flavone and flavonol biosynthesis and flavonoid biosynthesis pathways than the green pods. The KEGG pathway enrichment upregulated DAMs in the GP-S1_vs_GP-S2, and PP-S1_vs_PP-S2 were further analyzed, and the results showed that five flavonoids (quercetin 3-O-malonylglucoside, isoquercetrin, kaempferol 3-O-sophoroside, quercetin, and kaempferol) were co-upregulated in both GPs and PPs during the pod development, while two flavonoids ((±)-Naringenin and 5-p-Coumaroylquinic acid) were only upregulated in PPs (Table S4). This result indicated that the five co-upregulated flavonoid DAMs were essential metabolites for pea pod development, and the two upregulated flavonoid DAMs in PPs provided more intermediate metabolites for the purple coloration. These data suggested that the DAMs in the flavonoid biosynthesis pathway were the key metabolites underlying the variations in purple pods.

### 3.2. Transcriptome Analysis of Green and Purple Pods

A total of 12 libraries constructed from green and purple lines at the two different developmental stages of pods were subjected to an RNA-seq analysis to investigate the variation at the transcriptional level. A transcriptome sequencing of all of the samples yielded more than 4.0 Gb of high-quality sequencing data, and the Q30 (%) of each library was >97% (Table S5). The mapping rate of all of the libraries was 95.18%–96.52%, and unique mapped reads constituted >92%. Based on the high-quality sequencing data, the DEGs of each comparison were screened out under the threshold of log2|FC| > 1 and *p* < 0.05. 

**Figure 3:**
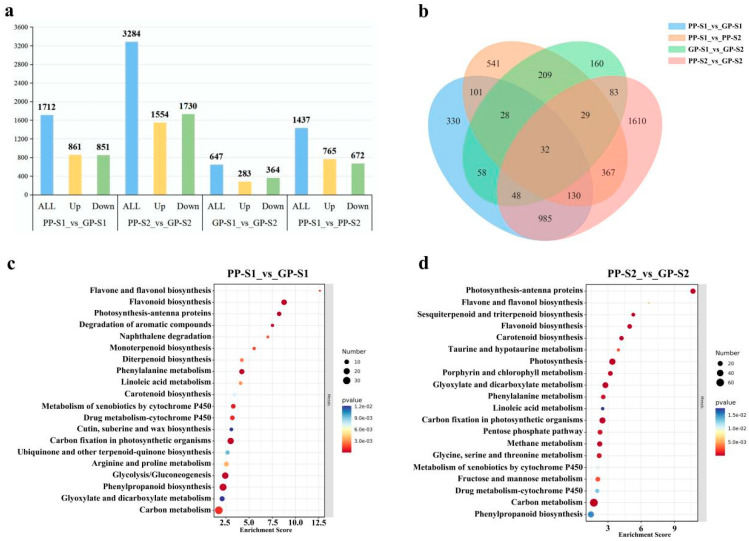
Transcriptome analysis between GPs and PPs at two developmental stages. (**a**). The statistics of differentially expressed genes (DEGs) identified from the transcriptome. (**b**). The Venn diagram of the DEGs in each comparison group. (**c**,**d**).The top 20 KEGG pathway enrichments of all DEGs in the comparison groups of PP-S1_vs_GP-S1 and PP-S2_vs_GP-S2.

As depicted in Figure 3a, there were 1712 DEGs (861 upregulated and 851 downregulated), 3284 DEGs (1554 upregulated and 1730 downregulated), 647 DEGs (283 upregulated and 364 downregulated), and 1437 DEGs (765 upregulated and 672 downregulated) in the four comparisons, respectively. The number of all of the DEGs in the two comparisons of different pod colors was higher than that in the other two comparisons of different pod stages, and the number of all DEGs in the PP-S2_vs_GP-S2 comparison was the highest compared with that in the other comparisons (Figure 3a). Furthermore, the number of DEGs in the PP-S1_vs_PP-S2 comparison was higher than that in the GP-S1_vs_GP-S2 comparison, suggesting that the purple pods required more genes involved in purple pigment coloration during pod development. A further Venn diagram analysis of the DEGs in the four comparisons revealed that there were 330, 541, 160, and 1610 unique DEGs in the PP-S1_vs_GP-S1, PP-S1_vs_PP-S2, GP-S1_vs_GP-S2, and PP-S2_vs_GP-S2 comparisons, respectively (Figure 3b). Moreover, 985 and 209 DEGs were shared in the comparisons of different pod colors and different pod developmental stages, respectively, suggesting that these DEGs are closely related to pod color variation and pod development, respectively. 

The top 20 KEGG pathway enrichments of DEGs in the PP-S1_vs_GP-S1 and PP-S2_vs_GP-S2 comparisons were analyzed (Figure 3c,d, Tables S6 and S7), which revealed two metabolic pathways related to pod color, including flavonoid metabolism and carotenoid metabolism. A further analysis of the KEGG pathway enrichments of upregulated and downregulated DEGs showed that the upregulated DEGs were enriched in flavonoid metabolism (ko00940, ko00941, ko00943, and ko00944), whereas the downregulated DEGs were enriched in carotenoid biosynthesis (ko00906) (Tables S6 and S7). These findings were consistent with the pod color variation.

### 3.3. Verification of RNA-Seq Data by RT-qPCR

**Figure 4:**
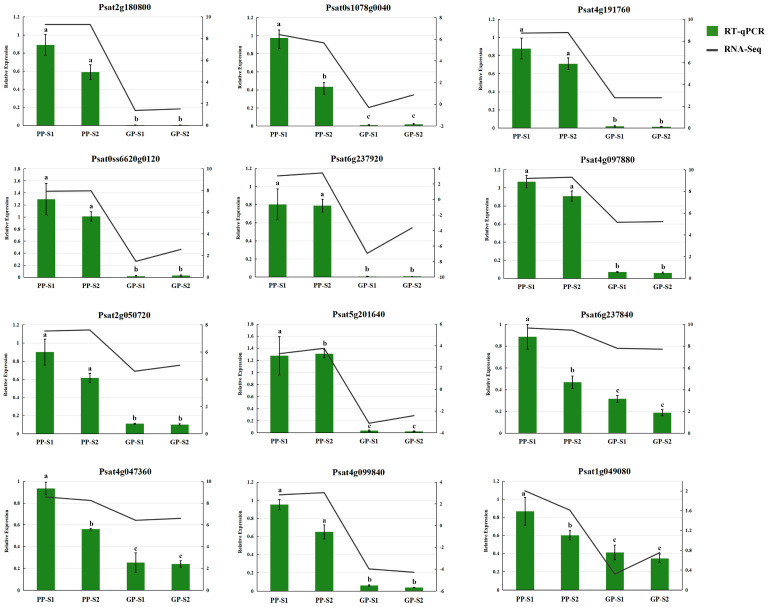
Validation of 12 genes randomly selected from DEGs by RT-qPCR. The ordinate indicates the relative expression, and the data are expressed as means ± SD, n = 3. The different lowercase letters represent statistically significant differences (*p* < 0.05, Duncan’s test).

The relative expression levels of the 12 DEGs involved in the flavonoid biosynthesis pathway were determined by a qRT-PCR to verify the RNA-seq data (Figure 4). The results indicated that the relative expression levels of the 12 DEGs were highly consistent with the expression level of RNA-seq, suggesting the reliability of the RNA-seq data. 

### 3.4. Analysis of DAMs and DEGs Involved in the Flavonoid Biosynthesis Pathway

**Figure 5:**
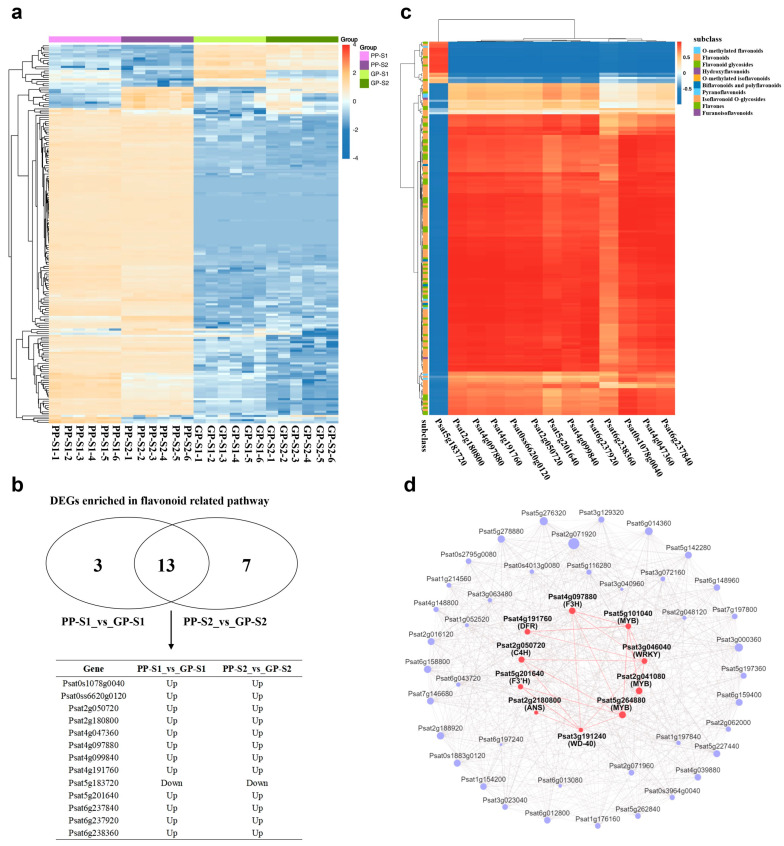
Analysis of DAMs and DEGs related to flavonoid metabolism. (**a**). Heatmap of all DAMs that were classified into flavonoid metabolites. (**b**). Venn analysis of co-regulated DEGs in PP-S1_vs_GP-S1 and PP-S2_vs_GP-S2 comparisons. (**c**). The heatmap of correlations between all flavonoid DAMs and 13 co-regulated DEGs involved in the flavonoid biosynthesis pathway. The red/blue lines represent positive/negative correlations. (**d**). The network of hub genes in the steelblue module identified from the WGCNA. The red dots represent the five structural genes involved in the flavonoid biosynthesis pathway, the WD-40 protein-encoding gene, and four transcription factor genes.

Based on the metabolome and transcriptome analyses, 180 flavonoid DAMs (Table S8) and 23 flavonoid-related DEGs (Tables S6 and S7) were identified from the KEGG pathway enrichment analysis, respectively. The heatmap showed that all 180 DAMs exhibited significantly differently accumulated patterns between the purple and green pods (Figure 5a), among which 152 and 19 flavonoid DAMs had significantly higher and lower accumulation in the purple pods at both developmental stages than that in the green pods, respectively. To clarify which flavonoid-related DEGs were co-regulated in the two stages of PPs, a Venn analysis was conducted and 13 co-regulated DEGs were identified (Figure 5b). Among the 13 co-regulated DEGs, 12 DEGs were co-upregulated and only 1 DEG was co-downregulated in the two comparisons. The co-downregulated gene *Psat5g183720* encoded the spermidine hydroxycinnamoyl transferase, indicating that this gene may negatively regulate the purple coloration of PPs. In addition, there were three and seven genes differentially expressed only in the PP-S1_vs_GP-S1 and PP-S2_vs_GP-S2 comparisons, respectively, indicating that different genes were involved in the coloring process at different pod development stages in PP. The correlation and significance between the 13 co-regulated DEGs and the 180 flavonoid DAMs were further analyzed (Figure 5c and Table S9). The results showed that the 12 co-upregulated DEGs were positively correlated with the accumulation of a large number of flavonoid DAMs and were also negatively correlated with the accumulation of 17 flavonoid DAMs. 

Moreover, a WGCNA was performed to investigate the gene regulatory network of flavonoid DAMs using the transcriptome data and 180 flavonoid DAMs. A total of 2688 genes were finally divided into 16 distinct modules based on the selected power value (30) (Figure S1a), wherein the steelblue module had the highest gene counts (Figure S1b). The clustering heatmap revealed the gene expression pattern of the steelblue module, wherein the results showed that the steelblue module correlated highly and positively with the accumulation pattern of the 180 flavonoid DAMs among the samples (Figure S1c). Gene networks were further constructed using the top 50 genes with the highest connectivity to identify the key hub genes within this module (Figure 4, Tables S10 and S11). In the steelblue module network, *Psat3g000360* (plasma membrane H+-ATPase), *Psat6g012800* (26S proteasome non-ATPase regulatory subunit 14 homolog), and *Psat5g262840* (strictosidine synthase protein family, which is an important protein in the alkaloid biosynthesis pathway) had the highest number of edges (27, 25, and 23 edges, respectively) (Table S11). Among the fifty hub genes, nine genes were related to the flavonoid biosynthesis pathway, of which five genes (*Psat2g180800*, *Psat4g191760*, *Psat5g201640*, *Psat2g050720*, and *Psat4g097880*) were identified together in the correlation analysis of flavonoid DEGs and DAMs (Figure 5c). The 50 hub genes in the steelblue module also included 5 transcription factor genes (*Psat5g264880*, *Psat6g014360*, *Psat2g041080*, *Psat3g046040*, and *Psat5g101040*) and the gene encoding the WD-40 protein (*Psat3g191240*).

### 3.5. Association Analysis of Transcriptome and Metabolome Involved in the Flavonoid Metabolic Network

**Figure 6:**
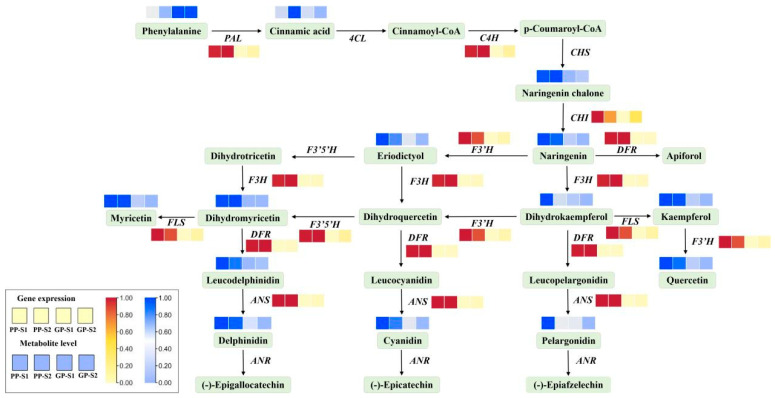
Joint analysis of the transcriptome and metabolome in the flavonoid biosynthesis pathway. The changes in metabolite accumulation and gene expression in the four samples were shown as heatmaps with blue and yellow boxes, respectively.

The combined analysis of the transcriptome and metabolome in the flavonoid biosynthesis pathway could systematically reveal the differences in gene expression and metabolite accumulation in the flavonoid biosynthesis pathway between the two pea accessions (Figure 6). In the flavonoid biosynthesis pathway, except for the *4CL*, *CHS* and *ANR* genes, 21 unigenes encoding nine enzymes were identified as DEGs and upregulated by 2.20- to 258.8-fold in the PP-S1_vs_GP-S1 comparison and by 0.41- to 216.79-fold in the PP-S2_vs_GP-S2 comparison (Table S12). As depicted in Figure 6, the expression levels of the nine structural genes, including *PAL*, *C4H*, *CHI*, *F3H*, *F3’H*, *F3’5’H*, *DFR*, *ANS* and *FLS* (Table S12), were significantly upregulated in the purple pod pea. For flavonoid metabolites, the accumulation trends of major DAMs, including naringenin chalcone, eriodictyol, naringenin, myricetin, dihydromyricetin, kaempferol, leucodelphinidin, delphinidin, cyaniding, and quercetin, were consistent with these upregulated DEGs and were highly reinforced by the higher gene expression levels in the purple pod pea at both developmental stages. The accumulation of three metabolites (cinnamic acid, dihydrokaempferol, and pelargonidin) peaked at one stage in the purple pod, and the phenylalanine level was the opposite of the trend of other flavonoid metabolites.

## 4. Discussion

Pod color is an important phenotypic trait of peas. Among the three different pod colors (green, yellow, and purple) in peas, the mechanism of color variation in the purple pod pea has not been explored. The purple pigmentation of plant tissue is generally rich in antioxidants and flavonoids, among which anthocyanin is an important compound responsible for the red–blue color [8,9,13]. In the present study, the potential regulatory mechanisms and networks of flavonoids and pod color differences between the purple pod and green pod peas at two different developmental stages were examined based on a comprehensive analysis of the transcriptome and metabolome to investigate the potential candidate gene resources for analyzing the molecular mechanism of pod color formation and genetic manipulation of pod color in peas. 

According to their different structures, flavonoids can be subdivided into different subgroups, including flavones, flavonols, flavanones, isoflavonoids, bioflavonoids, flavanols, anthocyanins, and chalcones [14]. For coloration, the composition and content of anthocyanins are important factors that determine the red, blue, and purple color in plants [14,15]. Cyanidin, delphinidin, malvidin, peonidin, petunidin, and pelargonidin are the six common anthocyanins [16]. Cyanidin and delphinidin are the major anthocyanins in the blue and purple flowers [17,18]. In this study, 2933 DAMs were identified between purple and green pod peas at two different developmental stages, of which 180 DAMs were flavonoid metabolites that belonged to the subgroups of flavones, flavonols, flavanones, isoflavonoids, flavanols, chalcones, and anthocyanins (Table S8). The anthocyanin species among the flavonoid DAMs included three cyanidins, six delphinidin, one peonidin, two petunidin, and one pelargonidin metabolites. Among the 13 anthocyanin metabolites, only 3 (petunidin 3-(6″-p-coumarylglucoside)-5-glucoside, peonidin 3-O-glucoside, and cyanidin 3-(6″-ferulylglucoside)-5-glucoside) showed high accumulation in the green pod peas at both developmental stages. These findings indicated that anthocyanins were the principal pigments in purple pod peas, and the high content of anthocyanin metabolites in the purple pod peas might play a major role in the purple color pigmentation.

Flavones and flavonoids have been reported to play a vital role as co-pigments in the process of color formation and development. Flavonoids and flavonols are not only the major branch products of the anthocyanin synthesis pathway, but they also combine with anthocyanins to form new pigment complexes and change the color of plants [19,20,21]. The deficient accumulation of the yellow-colored naringenin chalcone was found to be responsible for the pink coloration of the tomato fruit, indicating that the accumulation of flavones and flavonoids could also affect the color of plants [22]. In the present study, the analysis of flavonoid DAMs (Table S8) and the flavonoid biosynthesis pathway (Figure 6) indicated a significantly higher content of anthocyanin metabolites in the purple pod pea than in the green pod pea, and the accumulation of other flavone and flavonol compounds, including luteolin, kaempferol, and quercetin, was also significantly higher than that in the green pod pea. These flavonoid metabolites showed a high accumulation in the purple pod peas as co-pigment substances, which not only affected and assisted the formation of the purple color to a certain extent but also imparted a higher nutritional value to the purple pod peas. The metabolome analysis revealed that the significant accumulation of anthocyanins, flavones, and flavonol metabolites as chromogenic substances and co-pigments in the purple pod pea was the primary cause of the purple color pigmentation.

The biosynthesis pathway of flavonoid metabolites is complex and involves a series of structural and regulatory genes [16,23]. The flavonoid synthesis pathway is primarily completed by three stages of enzymatic reactions. The first stage is the phenylpropane synthesis pathway involving PAL, C4H, and 4CL, which finally catalyzes phenylalanine into 4-coumaryl coenzyme A. The second stage is the synthesis of dihydrokaempferol, dihydroquercetin, and dihydromyricetin catalyzed by CHS, CHI, F3H, F3ʹH, and F3ʹ5ʹH. The third stage is the specific anthocyanin synthesis involving DFR, ANS, ANR, and a series of transferases [24,25]. In the present study, the results of the combined metabolome and transcriptome analysis revealed that the significant accumulation of flavonoid metabolites in the purple pod pea was due to the significantly upregulated expression of a large number of structural genes in the flavonoid synthesis pathway (Figure 6).

Flavonoid biosynthesis is not only influenced by structural genes but also precisely regulated by transcription factors at the transcriptional level. The MBW complex, composed of the R2R3-MYB transcription factor, bHLH transcription factor, and WD-repeat proteins, is the major regulator of flavonoid and anthocyanin synthesis [26]. Among the MBW complex, the WD-40 protein is highly conserved and expressed in all plants, and it participates in the formation and stability of the MBW complex [27]. Typically, the expression of R2R3-MYB genes is also highly specific, with multiple copies of these genes present in plants, and these copies produce anthocyanins in different patterns or cell types [28,29,30], while bHLH transcription factor genes exhibit a wide range of biological functions in addition to regulating anthocyanin synthesis, and their expression is not limited to tissues with anthocyanin accumulation [31,32,33]. In the result of the WGCNA, the WD-40 protein-encoding gene (*Psat3g191240*) and four transcription factor genes, which were significantly upregulated in the purple pod pea and highly correlated with the five structural genes (jointly screened out from the WGCNA and the correlation of flavonoid-related DEGs and DAMs) in the flavonoid synthesis pathway, were screened out (Table S10). The four transcription factors included one WRKY transcription factor gene (*Psat3g046040*) and three MYB transcription factor family genes (*Psat5g264880*, *Psat2g041080*, and *Psat5g101040*). The WD-40 protein-encoding gene (*Psat3g191240*) highly correlated with *C4H* (*Psat2g050720*), *F3H* (*Psat4g097880*), *F3ʹH* (*Psat5g201640*), *DFR* (*Psat4g191760*), *ANS* (*Psat2g180800*), and two MYB transcription factor genes (*Psat5g264880* and *Psat2g041080*) in the hub gene network of the steelblue module (Figure 5d and Table S11). These findings showed that, compared with that in GP, the upregulation of the WD-40 protein-encoding gene in PPs could enable the MBW complex to more stably and efficiently upregulate the expression of the structural genes involved in the flavonoid synthesis pathway.

According to previous research, the WRKY transcription factor family is also regulated and involved in the flavonoid biosynthesis pathway [34]. The results of the BLAST sequence analysis showed that the WRKY transcription factor gene (*Psat3g046040*) identified in this study was homologous to *WRKY44*. In *Arabidopsis*, the *AtWRKY44* was confirmed to regulate the synthesis of proanthocyanin by activating the promoter of *TT8/bHLH042* gene [35]. In the present study, the WRKY transcription factor gene (*Psat3g046040*) highly correlated not only with the *F3H* (*Psat4g097880*) and *PAL* (*Psat3g072160*) genes but also with the MYB transcription factor gene (*Psat2g041080*) (Table S11). Based on these results, it was speculated that the WRKY transcription factor gene (*Psat3g046040*) upregulated the expression of *F3H* and *PAL* by binding to the MYB transcription factor gene (*Psat2g041080*) in the purple pod pea. The results of the BLAST sequence analysis showed that the MYB transcription factor genes (*Psat5g264880*, *Psat2g041080*, and *Psat5g101040*) identified from the WGCNA were homologous to *ATMYB113* (*AT1G66370*), *AtMYB6* (*AT4G09460*), and *AtMYB5* (*AT3G13540*), respectively. In *Arabidopsis* and other plants, *ATMYB113* was found to be physically associated with PHR1 (phosphorus signaling core protein, PHOSPHATE STARVATION RESPONSE1) to regulate anthocyanin accumulation in *Arabidopsis* seedlings under P-deficient conditions [36]. Furthermore, the homologous *AtMYB6* was identified as the R2R3-MYB transcription factor that promoted anthocyanin and proanthocyanin biosynthesis in *Populus tomentosa* [37]. Although *AtMYB5* was not involved in the biosynthesis of flavonoids in *Arabidopsis*, the homologous genes of *AtMYB5* in other plants were found to positively regulate the biosynthesis of anthocyanins and proanthocyanins [38,39,40,41]. In the present study, these three MYB transcription factor genes were significantly upregulated in PPs at both developmental stages and highly correlated with *C4H*, *F3H*, *F3ʹH*, *DFR*, and *ANS* genes, showing that these three MYB genes positively regulated the expression of the structural genes involved in the flavonoid synthesis pathway and the accumulation of flavonoid anthocyanin metabolites.

## 5. Conclusions

Metabolomics and transcriptomics data were used to systematically analyze the pod color variation between purple and green pod peas at two different developmental stages. A total of 180 flavonoid DAMs exhibited significantly different accumulation between the purple pod and green pod peas, of which 10 anthocyanin compounds showed a significantly high accumulation in purple pod peas, which primarily contributed to the purple color. At the transcriptome level, 23 DEGs related to flavonoid metabolism were identified, of which 21 DEGs were significantly upregulated in purple pod peas, including 12 structural genes involved in the flavonoid synthesis pathway. 13 DEGs were co-regulated in both stages of purple pod peas and highly correlated with the flavonoid DAMs. Furthermore, through a WGCNA, the WD-40 protein-encoding gene and four transcription factor genes were identified and found to be significantly upregulated in the purple pod pea, including one WRKY family and three MYB transcription factor family genes; these genes highly correlated with several structural genes in the flavonoid synthesis pathway. Overall, this study provides valuable information and a foundation for the mechanism of pod color variation and the responsible candidate genes in purple pod peas. 

## Data Availability

The transcriptomic sequencing data can be downloaded from the BioProject database (accession number: PRJNA1196006).

## Supplementary Materials

The following supporting information can be downloaded at: https://www.mdpi.com/article/10.3390/cimb47020094/s1.

## References

[1] McCrory M.A., Hamaker B.R., Lovejoy J.C., Eichelsdoerfer P.E. (2010). Pulse consumption, satiety, and weight management. Adv. Nutr..

[2] Zohary D., Hopf M. (1973). Domestication of pulses in the Old World: Legumes were companions of wheat and barley when agriculture began in the Near East. Science.

[3] Smýkal P., Coyne C.J., Ambrose M.J., Maxted N., Schaefer H., Blair M.W., Berger J., Greene S.L., Nelson M.N., Besharat N. (2014). Legume Crops Phylogeny and Genetic Diversity for Science and Breeding. Crit. Rev. Plant Sci..

[4] Ellis T.H., Hofer J.M., Timmerman-Vaughan G.M., Coyne C.J., Hellens R.P. (2011). Mendel, 150 years on. Trends Plant Sci..

[5] Shirasawa K., Sasaki K., Hirakawa H., Isobe S. (2021). Genomic region associated with pod color variation in pea (*Pisum sativum*). G3.

[6] Liu N., Lyu X.L., Zhang X.Y., Zhang G.W., Zhang Z.Q., Guan X.Y., Chen X.Y., Yang X.M., Feng Z.J., Gao Q. (2024). Reference genome sequence and population genomic analysis of peas provide insights into the genetic basis of Mendelian and other agronomic traits. Nat. Genet..

[7] Feng C., Chen B.Z., Hofer J., Shi Y., Jiang M., Song B., Cheng H., Lu L., Wang L.Y., Howard A. (2024). Genomic and Genetic Insights into Mendel’s Pea genes. bioRxiv.

[8] Yamuangmorn S., Prom-u-Thai C. (2021). The Potential of High-Anthocyanin Purple Rice as a Functional Ingredient in Human Health. Antioxidants.

[9] Zhao D., Tao J. (2015). Recent advances on the development and regulation of flower color in ornamental plants. Front. Plant Sci..

[10] Chayut N., Yuan H., Ohali S., Meir A., Yeselson Y., Portnoy V., Zheng Y., Fei Z., Lewinsohn E., Katzir N. (2015). A bulk segregant transcriptome analysis reveals metabolic and cellular processes associated with Orange allelic variation and fruit beta-carotene accumulation in melon fruit. BMC Plant Biol..

[11] Liu N., Zhang G., Xu S., Mao W., Hu Q., Gong Y. (2015). Comparative Transcriptomic Analyses of Vegetable and Grain Pea (*Pisum sativum* L.) Seed Development. Front. Plant Sci..

[12] Zhong X.Z., Yang M., Zhang X.Y., Fan Y.F., Wang X.S., Xiang C. (2024). Comparative analysis of transcriptome and metabolome explores the underlying mechanism of pod color variation in pea (*Pisum sativum* L.). J. Plant. Biochem. Biot..

[13] He J., Giusti M.M. (2010). Anthocyanins: Natural Colorants with Health-Promoting Properties. Annu. Rev. Food Sci. Technol..

[14] Panche A.N., Diwan A.D., Chandra S.R. (2016). Flavonoids: An overview. J. Nutr. Sci..

[15] Zhang Y., Butelli E., Martin C. (2014). Engineering anthocyanin biosynthesis in plants. Curr. Opin. Plant Biol..

[16] Mattioli R., Francioso A., Mosca L., Silva P. (2020). Anthocyanins: A Comprehensive Review of Their Chemical Properties and Health Effects on Cardiovascular and Neurodegenerative Diseases. Molecules.

[17] Qi Y.Y., Lou Q., Li H.B., Yue J., Liu Y.L., Wang Y.J. (2013). Anatomical and biochemical studies of bicolored flower development in Muscari latifolium. Protoplasma.

[18] Lou Q., Liu Y.L., Qi Y.Y., Jiao S.Z., Tian F.F., Jiang L., Wang Y.J. (2014). Transcriptome sequencing and metabolite analysis reveals the role of delphinidin metabolism in flower color in grape hyacinth. J. Exp. Bot..

[19] Bloor S.J., Falshaw R. (2000). Covalently linked anthocyanin-flavonol pigments from blue *Agapanthus* flowers. Phytochemistry.

[20] Mizuno T., Yabuya T., Kitajima J., Iwashina T. (2013). Identification of novel Cglycosylflavones and their contribution to flower colour of the Dutch iris cultivars. Plant Physiol. Biochem..

[21] Goto T., Kondo T. (1991). Structure and molecular stacking of anthocyanins—Flower color variation. Angew. Chem. Int. Ed. Engl..

[22] Ballester A.R., Molthoff J., de Vos R., Hekkert B., Orzaez D., Fernandez-Moreno J.P., Tripodi P., Grandillo S., Martin C., Heldens J. (2010). Biochemical and molecular analysis of pink tomatoes: Deregulated expression of the gene encoding transcription factor SlMYB12 leads to pink tomato fruit color. Plant Physiol..

[23] Tanaka Y., Sasaki N., Ohmiya A. (2008). Biosynthesis of plant pigments: Anthocyanins, betalains and carotenoids. Plant J..

[24] Springob K., Nakajima J.I., Yamazaki M., Saito K. (2003). Recent advances in the biosynthesis and accumulation of anthocyanins. Nat. Prod. Rep..

[25] Koes R., Verweij W., Quattrocchio F. (2005). Flavonoids: A colorful model for the regulation and evolution of biochemical pathways. Trends Plant Sci..

[26] Jaakola L. (2013). New insights into the regulation of anthocyanin biosynthesis in fruits. Trends Plant Sci..

[27] Van Nocker S., Ludwig P. (2003). The WD-repeat protein superfamily in Arabidopsis: Conservation and divergence in structure and function. BMC Genom..

[28] Espley R.V., Brendolise C., Chagne D., Kutty-Amma S., Green S., Volz R., Putterill J., Schouten H.J., Gardiner S.E., Hellens R.P. (2009). Multiple repeats of a promoter segment causes transcription factor autoregulation in red apples. Plant Cell.

[29] Espley R.V., Hellens R.P., Putterill J., Stevenson D.E., Kutty-Amma S., Allan A.C. (2007). Red colouration in apple fruit is due to the activity of the MYB transcription factor, MdMYB10. Plant J..

[30] Mahmoudi E., MOHAMMAD S.B., Yadollahi A., Hosseini E. (2012). Independence of color intensity variation in red flesh apples from the number of repeat units in promoter region of the MdMYB10 gene as an allele to MdMYB1 and MdMYBA. Iran. J. Biotechnol..

[31] Gonzalez A., Zhao M., Leavitt J.M., Lloyd A.M. (2008). Regulation of the anthocyanin biosynthetic pathway by the TTG1/bHLH/Myb transcriptional complex in Arabidopsis seedlings. Plant J..

[32] Jiang W., Liu T., Nan W., Jeewani D.C., Niu Y., Li C., Wang Y., Shi X., Wang C., Wang J. (2018). Two transcription factors TaPpm1 and TaPpb1 co-regulate anthocyanin biosynthesis in purple pericarps of wheat. J. Exp. Bot..

[33] Spelt C., Quattrocchio F., Mol J., Koes R. (2002). ANTHOCYANIN1 of petunia controls pigment synthesis, vacuolar pH, and seed coat development by genetically distinct mechanisms. Plant Cell.

[34] Schaart J.G., Dubos C., Romero De La Fuente I., van Houwelingen A.M., de Vos R.C., Jonker H.H., Xu W., Routaboul M.J., Lepiniec L., Bovy A.G. (2013). Identification and characterization of MYB-bHLH-WD40 regulatory complexes controlling proanthocyanidin biosynthesis in strawberry (*Fragaria x ananassa*) fruits. New Phytol..

[35] Xu W., Grain D., Le Gourrierec J., Harscoët E., Berger A., Jauvion V., Scagnelli A., Berger N., Bidzinski P., Kelemen Z. (2013). Regulation of flavonoid biosynthesis involves an unexpected complex transcriptional regulation of TT8 expression, in Arabidopsis. New Phytol..

[36] Li H., He K., Zhang Z., Hu Y. (2023). Molecular mechanism of phosphorous signaling inducing anthocyanin accumulation in Arabidopsis. Plant Physiol. Biochem..

[37] Wang L., Lu W., Ran L., Dou L., Yao S., Hu J., Fan D., Li C., Luo K. (2019). R2R3-MYB transcription factor MYB6 promotes anthocyanin and proanthocyanidin biosynthesis but inhibits secondary cell wall formation in Populus tomentosa. Plant J..

[38] Gonzalez A., Mendenhall J., Huo Y., Lloyd A. (2009). TTG1 complex MYBs, MYB5 and TT2, control outer seed coat differentiation. Dev. Biol..

[39] Liu C., Jun J.H., Dixon R.A. (2014). MYB5 and MYB14 play pivotal roles in seed coat polymer biosynthesis in *Medicago truncatula*. Plant Physiol..

[40] Akagi T., Ikegami A., Tsujimoto T., Kobayashi S., Sato A., Kono A., Yonemori K. (2009). DkMyb4 Is a Myb transcription factor involved in proanthocyanidin biosynthesis in *Persimmon* fruit. Plant Physiol..

[41] Deluc L., Barrieu F., Marchive C., Lauvergeat V., Decendit A., Richard T., Carde J.P., Merillon J.M., Hamdi S. (2006). Characterization of a grapevine R2R3-MYB transcription factor that regulates the phenylpropanoid pathway. Plant Physiol..

